# Retrospective analysis of the prognostic value of PD-L1 expression and ^18^F-FDG PET/CT metabolic parameters in colorectal cancer

**DOI:** 10.7150/jca.38689

**Published:** 2020-02-25

**Authors:** Hao Jiang, Rongjun Zhang, Huijie Jiang, Mingyu Zhang, Wei Guo, Jifeng Zhang, Xinglu Zhou, Wenbin Pan, Sheng Zhao, Ping Li

**Affiliations:** 1 Department of Radiology, The Second Affiliated Hospital of Harbin Medical University, Harbin, China; 2 Jiangsu Institute of Nuclear Medicine, Wuxi, China; 3 Department of Nuclear Medicine, Beijing Friendship Hospital, Affiliated to Capital Medical University, Beijing, China; 4 Department of Ultrasound, Harbin the First Hospital, Harbin, China; 5 Department of PET/CT, The Second Affiliated Hospital of Harbin Medical University, Harbin, China; 6 Department of PET/CT, Harbin Medical University Cancer Hospital, Harbin, China

**Keywords:** colorectal cancer, programmed cell death ligand 1 (PD-L1), ^18^F fluorodeoxyglucose (^18^F-FDG), positron-emission tomography, metabolism

## Abstract

**Background:** It has been rarely reported whether ^18^F-fluorodeoxyglucose (^18^F-FDG) uptake in colorectal cancer cells is associated with the expression of PD-L1. We performed a clinical pathology study to evaluate PD-L1 expression in patients undergoing surgical resection of colorectal cancer with preoperative ^18^F-FDG PET/CT imaging, with the aim of predicting the response of CRC patients to immune checkpoint inhibitors.

**Material and Methods**: A retrospective analysis of patients with CRC who underwent FDG-PET imaging before surgery was performed to measure the parameters of FDG-PET imaging: the maximum standardized uptake value (SUVmax), the metabolic tumor volume (MTV), and the total lesion glycolysis (TLG) were evaluated to determine whether each parameter was associated with clinical pathology. Tumor specimens were subjected to PD-L1 staining by immunohistochemistry. Analysis of whether there is a correlation between PD-L1 expression and ^18^F-FDG uptake parameters in CRC.

**Results**: PD-L1 expression level was significantly correlated with SUVmax, MTV3.0 and TLG3.0. Multivariate analysis showed that PD-L1 and TLG3.0 were independent predictors of poor DFS in patients with CRC (*P=*0.009; *P=*0.016), PD-L1 expression is closely related to the patient's lesion (TLG3.0) (*P*<0.01).

**Conclusion**: The results of this study indicate that there was a significant correlation between PD-L1 expression and TLG3.0 which suggested that FDG-PET could serve as a noninvasive tool to assess the tumor microenvironment and as a predictor of PD-L1 inhibitor activity to determine the optimal therapeutic strategy for CRC. High PD-L1 expression levels and high TLG3.0 are independent risk factors for DFS differences in CRC patients.

## Introduction

Colorectal cancer (CRC) is one of the major cancers worldwide^1^. According to Bray et al^2^, CRC is the third most commonly diagnosed cancer and the second most common cause of cancer-related death worldwide. Compared to traditional therapies, emerging cancer immunotherapy usually shows better tolerance and long-lasting effects^3^,^4^ . Especially the new checkpoint blockade therapy for PD-1 and its ligand PD-L1 has achieved unprecedented clinical effects in the treatment of the tumors 5,6. Studies have shown that the progression-free survival of PD-L1 antibody in the treatment of non-small cell lung cancer is significantly higher than the consolidation therapy of traditional radiotherapy and chemotherapy (16.8 months vs 5.6 months)^7^. Many clinical trials have demonstrated that immunotherapy significantly improves progression-free survival in patients. Remarkably, the grade 3 or 4 adverse events were evidently decreased compared to chemotherapy^8^. And, immunotherapy is suitable for PD-L1 positive patients^9^, so effective and non-invasive assessment of PD-L1 expression in patients' tumors is a problem to be solved. High expression of PD-L1 has been observed in a large number of solid tumors, including breast cancer^10^, NSCLC^11^, hepatocellular carcinoma^12^, renal cell carcinoma^13^, testicular cancer^14^. Some meta-analyses have shown that in many types of cancer, high PD-L1 expression is associated with adverse clinical and pathological outcomes, and the risk of death increases^15^-^17^. However, studies on the prevalence and prognosis of PD-L1 expression in CRC remain controversial. A study of another 454 CRC subjects showed that only 12% of patients had PD-L1 expression^18^. Higher expression of PD-L1 correlates with better prognosis of CRC patients^19^.

^18^F-FDG with positron emission tomography (PET) is a diagnostic method for distinguishing between benign and malignant lesions^20^. Some researchers reported that ^18^F-FDG PET can be used to monitor the efficacy of chemotherapy drugs and predict the outcome of any treatment for patients with multiple tumors^21^,^22^. Lopci et al^23^ suggest that FDG-PET could serve as a noninvasive tool to assess the tumor microenvironment and therefore predict benefit from PD-L1 blockade or other immunotherapy strategies. However, the relationship between the immune environment, including ^18^F-FDG uptake and PD-L1 expression in colorectal cancer are unclear.

In the present study, we aimed to investigate whether PD-L1 expression correlates with ^18^F-FDG uptake parameters to assess whether ^18^F-FDG PET/CT imaging can be used to predict PD-L1 expression in colorectal cancer. The maximum standard uptake value (SUVmax) only reflects the extent of glucose utilization in the tumor and does not accurately assess the metabolic activity of the tumor as a whole. MTV and TLG provide additional information on intratumoral biological variation. These parameters appear to be reliable indicators for assessing tumor metabolism and have become effective markers for the diagnosis and prognosis of several cancers, including lung and cervical cancer^24^-^27^. Therefore, this paper combined MTV, TLG and SUVmax to analyze the relationship between ^18^F-FDG metabolic parameters and CRC immune microenvironment (PD-L1 expression), and to evaluate the expression level of PD-L1 in patients undergoing surgical colorectal cancer resection with ^18^F-FDG PET/CT before surgery. To the best of our knowledge, this is a small number of studies providing ^18^F-FDG PET/CT imaging potential evidence for PD-L1 expression in colorectal cancer.

## Material and Methods

### Patients

From March 2016 to March 2019, patients with CRC were enrolled in the study; all patients underwent ^18^F-FDG PET/CT imaging at the Affiliated Tumor Hospital of Harbin Medical University, followed by tumor resection.

The inclusion criteria were as follows: ^18^F-FDG PET/CT images were obtained before given adjuvant therapy, and colorectal cancer (enlarged) radical resection was performed after imaging; histopathological examination of surgical specimens confirmed the diagnosis of colorectal cancer; complete case records available, including age, gender, tumor size, TNM stage, and degree of differentiation; available immunohistochemical staining tissue specimens. Exclusion criteria were as follows: history of adjuvant therapy before ^18^F-FDG PET/CT imaging; the pathological tissue specimens of immunohistochemical analysis are insufficient in size; incomplete clinical and pathological data. Complete medical records are available to all patients, including patient clinical data and follow-up information.

Data on clinical pathology was extracted from the medical records. Overall survival and disease-free survival were extracted from the medical records and telephone follow-up. Disease-free survival (DFS) was defined as the time from surgery to tumour recurrence, while overall survival (OS) was defined as the time from surgery to death from any cause.The study was approved by the Ethics Committee of the Affiliated Tumor Hospital of Harbin Medical University and complies with the revised principles of the 2013 Helsinki Declaration.

### Immunohistochemical staining

PD-L1 immunohistochemical staining was performed according to previous experimental procedures^28^,^29^. The PD-L1 rabbit monoclonal antibody (Abeam, ab205921; UK) was used at a primary antibody concentration of 1:100. The pathological specimens were washed three times with PBS, citrate antigen was repaired, goat serum was blocked for 20 min, and the primary antibody was incubated at 4 °C overnight. On the next day, the corresponding secondary antibody (Zhongshan Jinqiao) was added dropwise to the slide and incubated for 20 min at room temperature. Then, hematoxylin was counterstained and observed under a microscope (magnification: 200; Olympus BX53; Japan). When the dyeing intensity and position were optimal, it was rinsed with tap water, and then dehydrated and sealed with a neutral resin. When membrane staining was observed, the expression of PD-L1 was considered positive. Semi-quantitative scoring method for PD-L1:1 = <1%, 2= 1-5%, 3=6-10%, 4= 11-25%, 5= 26-50% and 6= > 50% positive cells. Tumors with a score greater than or equal to 3 were rated as high expression. At least two investigators examined the tissue sections blindly. If there is any discrepancy, the two investigators will simultaneously evaluate the slices until their assessment reaches a final consensus. The investigators were blinded to the patient outcomes.

### ^18^F-FDG PET/CT imaging and data analysis

All patients were fasted for at least 8-12 hours prior to PET imaging and orally laxative (complex polyethylene glycol electrolyte) to clear the intestine for enema in patients with intestinal obstruction. This study did not use intravenous drugs to inhibit intestinal peristalsis. PET imaging was performed using a PET/CT scanner (Discovery ST: GE Medical systems, Milwaukee, WI, USA). Before the injection of ^18^F-FDG, the patient measured the blood glucose through the fast blood glucose test paper, and controlled the blood glucose level to below 8mmol/L. After the injection, the whole body PET/CT imaging was performed for about 60 minutes. The tube voltage is 120kV, the tube current is 200mAs, and the slice thickness is 3.75mm. PET collection uses 3D mode PET scanning, 2.5min/bed, and generally scans 6-8 beds. Image recombination reconstructs images using the ordered subset maximum expectation method. All ^18^F-FDG PET/CT images were reviewed by two experienced senior physicians who were unaware of the patient's clinical history and laboratory results. They manually delineate the region of interest (ROI) in colorectal cancer lesions, avoiding heterogeneous regions such as hemorrhage, cystic changes, and necrosis, and measuring SUVmax and mean standardized uptake value (SUVmean) at the most concentrated level of concentration. Inconsistent results were resolved through consensus review. After the PET/CT image is transmitted into the GE PET-VCAR post-processing software, the ROI is automatically outlined by the software using the fixed threshold method and the SUVmax2.5, SUVmax3.0 and SUVmax3.5 as the threshold values on the PET image, manual adjustment from the axial、coronal and sagittal directions to determine the optimal boundary of the primary lesion, then the software automatically generates MTV (2.5, 3.0, 3.5) within the ROI and calculates the corresponding TLG (2.5, 3.0, 3.5) Value. Calculated as follows:


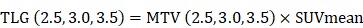


### Statistical analysis

Statistical significance was expressed as *P <0.05*. χ^2^ or Fisher exact test is used to test whether there was a difference between the clinical data of the ^18^F-FDG uptake parameters in the PET/CT imaging group. The receiver operating characteristic (ROC) curve analysis was used to examine the possibility that each parameter of ^18^F-FDG uptake distinguishes between high and low expression of PD-L1, determine the optimal cutoff value for each parameter, and calculate sensitivity and specificity. The correlation between the parameters of ^18^F-FDG uptake and PD-L1 was assessed using the Spearman correlation coefficient test. The Kaplane Meier method was used to estimate survival as a function of time and to analyze survival differences by Log-rank. Statistical analysis was performed using SPSS software (version 19.0).

## Results

### Patient demographics

**Figure 1:**
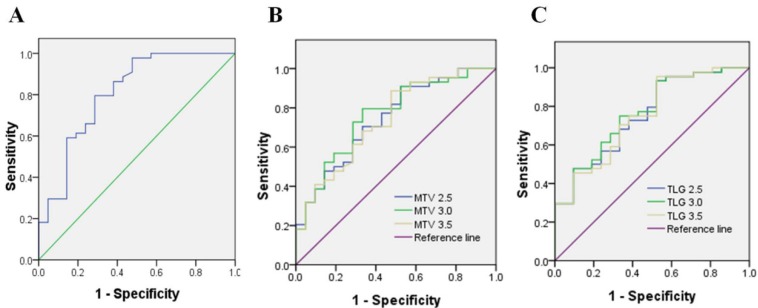
A: SUV value parameter to identify the diagnostic performance of PD-L1 high and low expression, the best cutoff value is 15.46. B、C: MTV and TLG parameters identify the diagnostic efficacy of PD-L1 high and low expression, and MTV 3.0 and TLG 3.0 have a larger area under the curve, which is better diagnostic efficiency.

According to the inclusion and exclusion criteria, a total of 65 patients (37 males, 28 females, age range 23-79 years, median age 60 years) were enrolled in the study. The patients' clinicopathological features are listed in Table 1. The best SUVmax cutoff value was determined by ROC curve analysis to be 15.46, the sensitivity was 79.5%, and the specificity was 71.4%(Figure 1). Patients with a SUVmax greater than 15.46 were defined as high SUVmax uptake. High SUVmax uptake was found in 43 (66%) of 65 patients. The relationship between SUVmax and patient clinicopathological features is shown in Table 2. High SUVmax was significantly associated with tumor differentiation (*P*=0.025), tumor size (*P*=0.011), TNM stage (*P*=0.006), and tumor vascular invasion (*P*=0.000). Similarly, the ROC curve analysis confirmed that MTV3.0 and TLG3.0 had the highest diagnostic performance for PD-L1 high and low expression, the best cut off values were 28.05 and 182.9, respectively, the sensitivity was 79.5% and 75%, respectively, and the specificity was 66.7% and 66.7%(Figure 1). High MTV3.0 was found in 42 (65%) of 65 patients, and high TLG3.0 was found in 40 (62%). The relationship between MTV 3.0, TLG 3.0 and patient clinicopathological features is shown in Table 3. High MTV3.0 and TLG3.0 were significantly associated with tumor differentiation, tumor size, lymph node metastasis, tumor TNM stage, and tumor lymphatic and vascular invasion.

### Immunohistochemistry results

**Figure 2:**
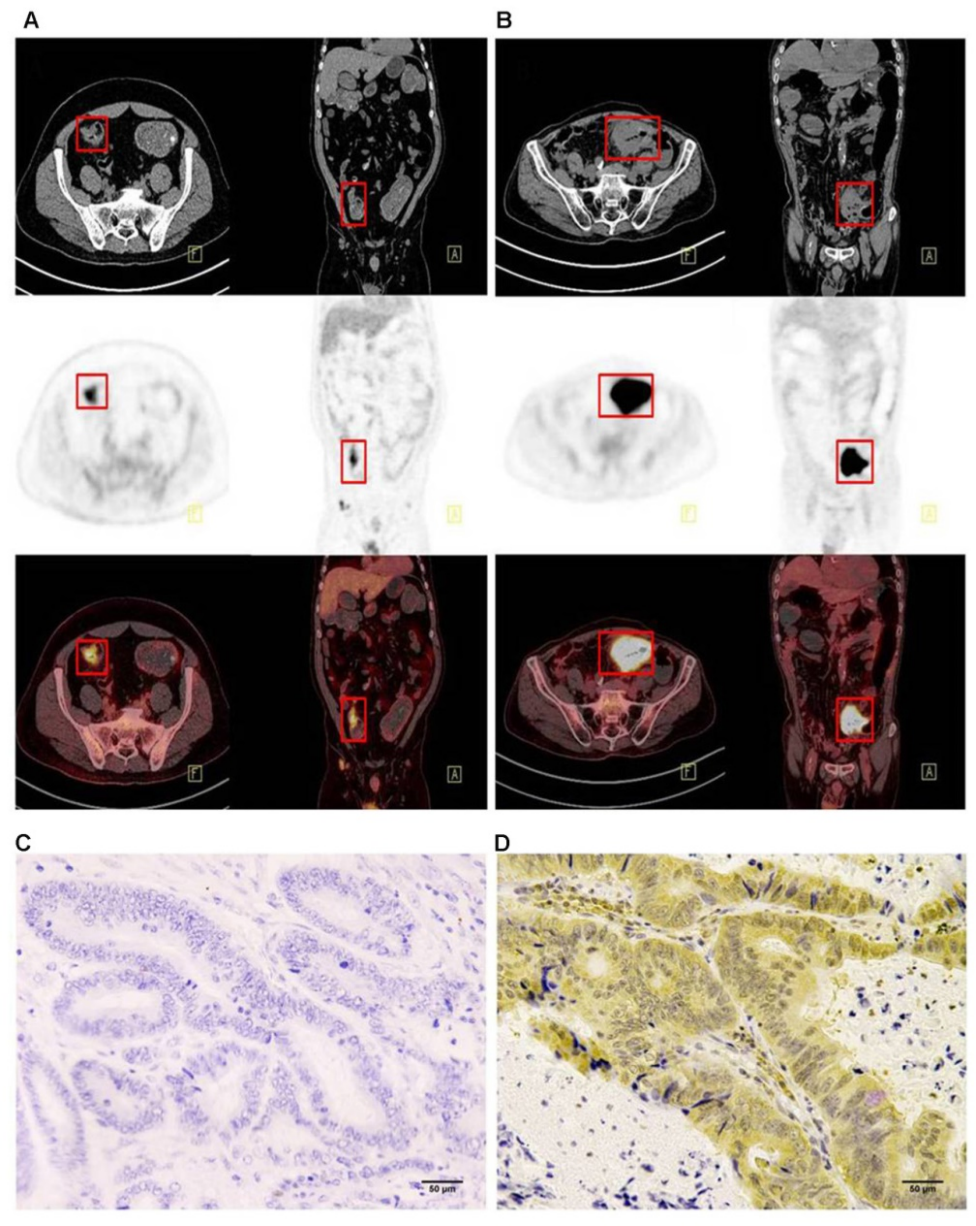
Representative imaging of immunohistochemical staining with PD-L1 expression and ^18^F-FDG PET with high ^18^F-FDG (B) (D) and low ^18^F-FDG accumulation (A) (C): The maximum standardised uptake values were 9.09 (A) and 39.01 (B) (red frame). The immunostaining pattern of PD-L1 was membrane, and the cases with scoring of 2 (C) and scoring of 6 (D) were presented. PD-L1, programmed death ligand-1; ^18^F-FDG, 2-Deoxy-2-[fluorine-18] fluoro-D-glucose; PET, positron emission tomography.

**Figure 3:**
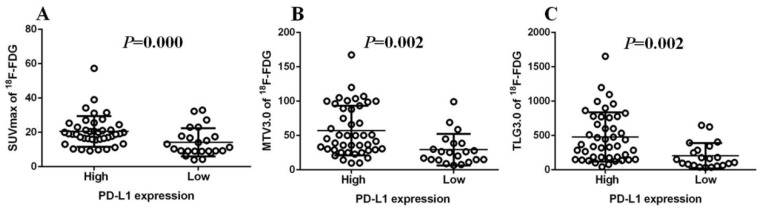
Comparison of SUVmax、MTV3.0 and TLG3.0 according to PD-L1 expression: SUVmax was significantly higher in patients with high PD-L1 expression than that in those with low expression (*P*=0.000) (A); MTV3.0 was significantly higher in patients with high PD-L1 expression than that in those with low expression (*P* =0.002) (B); TLG3.0 was significantly higher in patients with high PD-L1 expression than that in those with low expression (*P*=0.002) (C). PD-L1, programmed death ligand-1; SUVmax, standardised uptake value; MTV, metabolic tumor volume; TLG, total lesion glycolysis.

Immunohistochemistry was performed using the main sites of 65 CRC. A representative image of PD-L1 high and low expressions are shown in Figure 2. PD-L1 immunostaining is mainly localized in the plasma membrane of cancer cells^30^. The high PD-L1 expression rate was 68% (44/65). The relationship between PD-L1 and patient clinicopathological features is shown in Table 1. High expression of PD-L1 was significantly associated with tumor size (*P*=0.001), lymph node metastasis (*P*=0.034), tumor TNM stage (*P*=0.017), and tumor vascular invasion (*P*=0.020). And based on the comparison of SUVmax, MTV3.0 and TLG3.0 expressed by PD-L1, SUVmax, MTV3.0 and TLG3.0 values were significantly higher in patients with CRC with high PD-L1 expression than those with low expression; the difference was statistically significant (Figure 3).

### Correlation between PD-L1 expression and various parameters of ^18^F-FDG uptake

The correlation between SUVmax, MTV 3.0 and TLG 3.0 and PD-L1 is listed in Table 4. Spearman analysis showed that PD-L1 expression was significantly associated with higher SUVmax, MTV3.0 and TLG3.0 (Table 4) (rho=0.50, *P*<0.0001; rho=0.42, *P*=0.0004; rho=0.43, *P*=0.0003).

### Univariate and multivariate survival analysis

**Figure 4:**
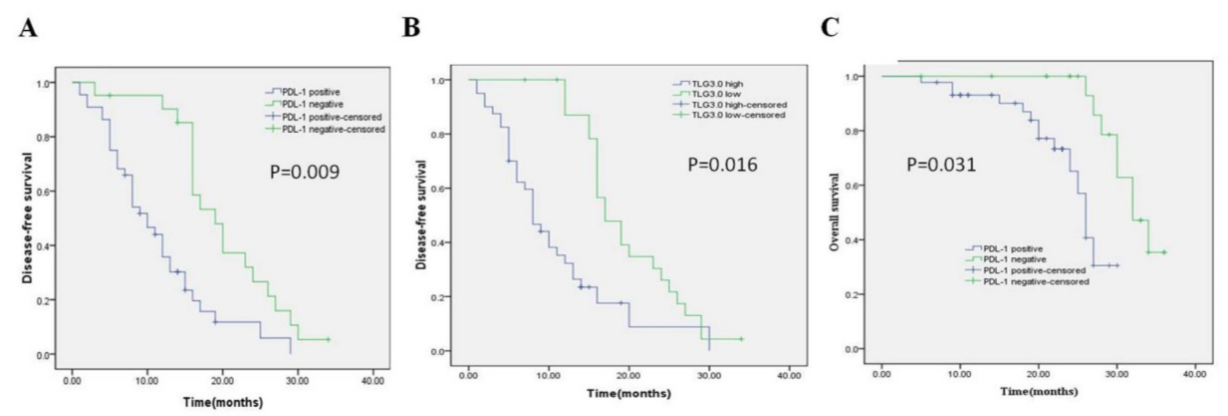
Kaplan-Meier survival curves for patients with CRC. (A) DFS curves for patients with negative PD-L1 expression and patients with positive PD-L1 expression. (B) DFS curves for patients with a low TLG3.0 and patients with a high TLG3.0. (C) OS curves for patients with negative PD-L1 expression and patients with positive PD-L1 expression.

Univariate and multivariate analyses were performed on all patients (Table 5, Table 6). In the univariate analysis, patients with high PD-L1 expression showed shorter DFS (*P* = 0.000) and OS (*P* = 0.002) than patients with low PD-L1 expression. Patients with higher SUVmax, MTV3.0, and TLG3.0 had significantly lower DFS (*P* = 0.004, *P* = 0.001, *P* = 0.000) and OS (*P* = 0.048, *P* = 0.003, *P* = 0.004) than patients with lower SUVmax, MTV 3.0 and TLG3.0. In addition, tumor differentiation, tumor size, and vascular invasion were also identified as independent risk factors for DFS; independent risk factors for OS were vascular invasion, PD-L1, SUVmax, MTV3.0, and TLG3.0. Based on the results of the univariate log-rank test, we screened variables with *P* < 0.05. Multivariate analysis showed that PD-L1, TLG3.0 were significant independent predictors of DFS, and independent predictors of OS were PD-L1 expression and tumor vascular invasion (Table 6). Figure 4 shows KaplaneMeier survival curves for patients with high and low TLG3.0 and PD-L1 expression.

## Discussion

This retrospective study assessed the clinicopathological significance of PD-L1 expression correlated with SUVmax, MTV3.0, and TLG3.0 in surgically resected CRC tissue. We found that SUVmax, MTV3.0, and TLG3.0 were significantly higher in PD-L1 high-expression colorectal cancer than in PD-L1 low-expression colorectal cancer. These results indicate that the expression level of PD-L1 is significantly correlated with tumor metabolism, metabolic volume and total glycolysis. The maximum standard uptake value (SUVmax) of ^18^F-FDG-PET is widely used in clinical practice due to its simplicity^31^ . However, it reflects the degree of glucose utilization of the tumor and does not accurately assess the metabolic activity of the tumor as a whole. MTV and TLG provide additional information on intratumoral biological variation. The relationship between PD-L1 expression in colorectal cancer and multiple parameters such as SUVmax, MTV and TLG, and the possible underlying mechanisms are still unclear. To the best of our knowledge, this is a small study analyzing the relationship between multi-parameters of ^18^F-FDG uptake and PD-L1 expression in colorectal cancer.

Immunotherapy against PD-1/PD-L1 has been successfully used to treat a variety of malignancies including, but not limited to, melanoma, lung cancer, kidney cancer, and bladder cancer^32^-^35^ . However, the clinical features associated with the benefits of immunotherapy remain largely unknown and identifying patients who may benefit from PD-1/PD-L1 blockade, while excluding those who may not respond to treatment remains unresolved challenges. By immunohistochemical assessment, expression of PD-L1 has been tested as a predictive biomarker for response to checkpoint inhibitors in colorectal cancer. However, this procedure requires invasive biopsy, so alternative non-invasive strategies for predicting PD-L1 expression, such as PET/CT imaging, and provides information for anti-PD-L1 antibody treatment strategies in colorectal cancer patients, and will be of great value for cancer immunotherapy. In this study, PD-L1 was highly expressed in 68% of CRC patients. In particular, the expression of PD-L1 is closely related to SUVmax, MTV3.0, and TLG3.0, which may be an important indicator for predicting the poor prognosis of CRC patients with high ^18^F-FDG intake related parameters. This retrospective study evaluated a significant association between ^18^F-FDG uptake-related parameters (SUVmax, MTV3.0, TLG3.0) and PD-L1 expression in surgically resected CRC. The expression rate of this positive PD-L1 was similar to that of the previous CRC study, and the positive expression of PD-L1 was found in 81.8% of metastatic CRC, which was more common than in primary CRC (40.9%; P = 0.012)^36^. These results indicate that the expression level of PD-L1 is significantly correlated with tumor metabolism, metabolic volume and total glycolysis. SUVmax, MTV3.0, and TLG3.0 values were significantly higher in patients with high PD-L1 expression than in low-expression patients. Previous studies have shown that FDG-PET could serve as a noninvasive tool to assess the tumor microenvironment and therefore predict benefit from PD-1/PD-L1 blockade or other immunotherapy strategies^23^. The results are consistent with the results of our study. In multivariate analysis, PD-L1 and TLG3.0 were identified as independent predictors of worse DFS in CRC patients, and DFS in patients with high PD-L1 and TLG3.0 were significantly lower. In addition, multivariate analysis showed that increased PD-L1 expression was significantly associated with low OS in CRC patients, and the results were consistent with previous studies. Wu et al^37^ conducted two studies demonstrating that positive PD-L1 expression is associated with a poor 5-year OS CRC. The high expression of PD-L1 is associated with low OS in CRC, high expression of PD-L1 is an independent predictor of colorectal cancer prognosis, PD-L1 knockdown can inhibit cell proliferation, migration and invasion^38^ . It can be seen that the expression of PD-L1 is associated with the prognosis of colorectal cancer. Patients with tumors with high expression of PD-L1 had poorer DFS and OS than patients with tumors with low expression of PD-L1.

Studies have supported our view that increased PD-L1 expression allows tumor cells to evade host immune surveillance and promote disease progression^39^ . In contrast, the results of another previous study showed that low PD-L1 expression was significantly associated with tumor recurrence and poor prognosis in stage III CRC^40^. In 2015, Le et al^41^ found that a higher tumor mutational burden (TMB) has been shown to correlate with clinical benefit from immunotherapy within colorectal cancer. In 2019, Yarchoan et al^42^ also found PD-L1 expression and TMB may each inform the use of immunotherapy, and identified new opportunities for therapeutic development. It can be seen that high expression of PD-L1 is associated with favorable and unfavorable prognosis in different studies. Moreover, the specificity of immunotherapy of tumor is not only related to the expression of PD-L1, but also may be related to TMB. Some of these findings may be due to differences in antibody selection, determination of thresholds used to determine PD-L1 positivity, and heterogeneity within the tumor. Further analyses are in need to further assess the prognostic value of PD-L1 for CRC patients receiving immunotherapy.

Due to its high repeatability and availability, SUVmax is currently the most commonly used ^18^F-FDG PET/CT parameter for diagnosis, performing TNM staging and monitoring treatment. Most studies have shown that SUVmax in CRC is associated with prognostic assessment. Marcus et al^43^ said that SUVmax is higher in patients with poor prognosis in CRC. Unlike SUVs, PET/CT metabolic parameters obtained by measuring high metabolic activity areas of tumors can provide volume and metabolic information for highly metabolized tumor cells. It has been reported in several malignant tumors (including pancreatic cancer, cervical cancer)^44^,^45^ that the PET/CT metabolic parameters represented by MTV and TLG are independent prognostic factors. However, the prognostic significance of MTV and TLG for CRC remains unclear. Therefore, this study evaluated the significance of SUVmax, MTV, and TLG for prognostic factors in patients with CRC. In a multivariate analysis, we found that tumor high TLG3.0 was an independent predictor of poor DFS in patients with CRC. It is indicated that TLG3.0 has important predictive value for disease-free survival time of CRC patients. Previous studies have shown that MTV and TLG in PET/CT are reliable biomarkers for CRC diagnosis. Using these parameters, a more accurate preoperative diagnosis of the CRC can be performed^46^ .

We also studied the biological characteristics of tumors with high and low FDG uptake. Our study showed that there were differences in tumor differentiation, tumor size, lymph node metastasis, TNM stage, and tumor vascular invasion in patients with different SUVmax, MTV3.0, and TLG3.0 CRC, and the difference was statistically significant. New therapies based on PD-1/PD-L1 inhibitors are known to exhibit impressive anti-tumor activity in tumor patients such as NSCLC and have recently become standard therapies for NSCLC^47^ . It can be seen that in this era of personalized medicine, clinicians face the following important questions: How do doctors identify patients who are more likely to benefit from anti-PD-1/PD-L1 treatment? Recent studies have shown that PD-L1 overexpression has become a useful predictor of poor CRC prognosis, and PD-L1 overexpression may prove valuable for screening candidates for anti-PD-1/PD-L1 therapy^38^ . In this study, we investigated the relationship between PD-L1 expression and clinicopathological factors. Our results showed that there were differences in tumor TNM stage, tumor size, lymph node metastasis and tumor vascular invasion in patients with different PD-L1 expression levels, and the difference was statistically significant (*P* <0.05). That is, patients with worse clinical pathological features have higher PD-L1 expression in patients with better clinical pathology, indicating that PD-L1 expression can be used as a marker of disease progression. These patients may benefit more from PD-L1 immunological checkpoint inhibitors than other patients. In addition, this study investigated whether SUVmax, MTV3.0, and TLG3.0 can predict PD-L1 expression in tumor tissues of patients with CRC. We observed a positive correlation between SUVmax, MTV3.0, and TLG3.0 and PD-L1 expression. Our results suggest that FDG-PET can be used as a non-invasive tool for assessing PD-L1 expression, thus predicting the benefits that may be derived from the PD-1/PD-L1 pathway-targeted immunotherapy.

This study has some limitations. First, studying the limited sample size of the cohort may reduce the statistical power of data analysis. Secondly, the deviation of the material selection, the different periods of tumor growth, different storage time and environment, and even the heterogeneity inside the tumor will make the PD-L1 expressed by the tumor bias. Third, different studies use different antibodies, and antibody selection may affect the results of the study. In this study, we used the clone 28-8 antibody, which has been used in clinical trials of nivolumab. In future studies, it may be necessary to validate different anti-PD-L1 antibodies in the same CRC sample.

## Conclusion

In conclusion, our results indicate that there is a significant correlation between TLG3.0 and PD-L1 expression levels, suggesting a potential role for ^18^F-FDG PET/CT to characterize the tumor microenvironment and select CRC patients' candidate to checkpoint inhibitors. In patients with high TLG3.0, PD-L1 is an independent predictor of low postoperative DFS; therefore, the accumulation of total glycolysis in CRC patients may help to predict the predictive effect of PD-L1 expression. Moreover, high PD-L1 expression is also an independent predictor of poor OS in CRC patients, further confirming that PD-L1 can be used as a target for immunotherapy in CRC patients. The future directions is to clarify why immunological checkpoint molecules such as PD-L1 can affect the glycolysis of tumor cells, and further explore the value of ^18^F-FDG PET/CT in predicting anti-PD-1/PD-L1 therapy response, it plays a key role in selecting the best treatment strategy for CRC patients.

## Figures and Tables

**Table 1:** Patients' Characteristics

Characteristics	N=65
Age	
≤60 years/>60 years	37/28
Gender	
Male/female	37/28
Tumor location	
Rectum/colon	13/52
Differentiation	
Well/ Moderate or poor	26/39
Tumor size	
≤3cm/>3cm	28/37
Lymph node metastasis	
Positive/ Negative	48/17
TNM stage	
I-II/III-IV	21/44
Lymphatic permeation	
Positive/ Negative	29/36
Vascular invasion	
Positive/ Negative	29/36
PD-L1	
High/ Low	44/21

**Table 2:** Patient's demographics according to SUVmax and PD-L1 expression.

Variables		PD-L1			SUVmax	
	High (n=44)	Low (n=21)	*P* value	High (n=43)	Low (n=22)	*P* value
Age						
≤60years/>60 years	24/20	13/8	0.575	23/20	14/8	0.434
Gender						
Male/female	24/20	13/8	0.575	23/20	14/8	0.434
Tumor location						
Rectum/colon	7/36	6/16	0.336	11/32	2/20	0.190
Differentiation						
Well/ Moderate or poor	14/30	12/9	0.051	13/30	13/9	0.025*
Tumor size						
≤3cm/>3cm	13/31	15/6	0.001*	15/28	15/7	0.011*
Lymph node metastasis						
Positive/ Negative	36/8	12/9	0.034*	36/7	12/10	0.011*
TNM stage						
I-II/III-IV	10/34	11/10	0.017*	9/34	12/10	0.006*
Lymphatic permeation						
Positive/ Negative	21/23	8/13	0.465	22/21	7/15	0.138
Vascular invasion						
Positive/ Negative	24/20	5/16	0.020*	26/17	3/19	0.000*

* P < 0.05. *P* < 0.05 is considered statistically significant. PD-L1, programmed death ligand-1.

**Table 3:** Patient's demographics according to MTV3.0 and TLG3.0.

Variables		MTV3.0			TLG3.0	
	High (n=42)	Low (n=23)	*P* value	High (n=40)	Low (n=25)	*P* value
Age						
≤60years/>60years	24/18	13/10	0.961	22/18	15/10	0.692
Gender						
Male/female	22/20	15/8	0.318	22/18	15/10	0.692
Tumor location						
Rectum/colon	9/33	4/19	0.758	9/31	4/21	0.524
Differentiation						
Well/ Moderate or poor	9/33	14/9	0.000*	9/31	17/8	0.000*
Tumor size						
≤3cm/>3cm	10/32	15/8	0.001*	11/29	17/8	0.001*
Lymph node metastasis						
Positive/ Negative	37/5	15/8	0.049*	33/7	15/10	0.045*
TNM stage						
I/II/III/IV	4/2/3/33	12/3/0/8	0.000*	3/1/4/32	13/4/2/6	0.000*
Lymphatic permeation						
Positive/ Negative	24/18	5/18	0.006*	24/16	5/20	0.002*
Vascular invasion						
Positive/ Negative	29/13	4/19	0.000*	26/14	3/22	0.000*

* P < 0.05. *P* < 0.05 is considered statistically significant.

**Table 4:** Correlation with PD-L1 expression and FDG metabolic parameter.

Variables		PD-L1	
	rho	95%CI	*P* value
All patients(n=65) SUVmax	0.50	0.28 to 0.67	<0.01*
MTV3.0 TLG3.0	0.42 0.43	0.19 to 0.61 0.20 to 0.62	<0.01* <0.01*

* P < 0.05. *P* < 0.05 is considered statistically significant. PD-L1, programmed death ligand-1; CI, confidence interval.

**Table 5:** Univariate and multivariate analysis of prognostic factors for disease-free survival.

Factor	Univariate analysis		Multivariate analysis	
	HR (95%CI)	*P* value	HR (95%CI)	*P* value
Age(>60vs≤60)	0.891(0.517-1.537)	0.667		
Gender(male vs female)	0.896(0.516-1.558)	0.688		
Differentiation(well vs moderate/poor)	0.449(0.250-0.804)	0.004*	0.530(0.202-1.394)	0.198
Tumor size(>3cm vs ≤3cm)	2.071(1.170-3.666)	0.008*	0.733(0.261-2.060)	0.556
Lymph node metastasis (yes vs no)	1.838(0.955-3.536)	0.054		
TNM stage (IV-III vs II-I)	0.587(0.330-1.042)	0.055		
Tumor location(rectum vs colon)	1.049(0.492-2.234)	0.898		
Ly(positive vs negative)	1.427(0.829-2.458)	0.181		
Vascular invasion(positive vs negative)	2.396(1.295-4.433)	0.003*	1.162(0.577-2.340)	0.675
PD-L1 (positive vs negative)	2.887(1.566-5.325)	0.000*	2.914(1.307-6.497)	0.009*
SUVmax (high vs low)	2.279(1.268-4.098)	0.004*	1.194(0.543-2.626)	0.658
MTV3.0(high vs low)	2.426(1.364-4.315)	0.001*	0.323(0.078-1.345)	0.121
TLG3.0(high vs low)	2.853(1.596-5.101)	0.000*	5.784(1.388-24.094)	0.016*

*P < 0.05 is considered statistically significant, calculated with continuous variable.CI, confidence interval; Ly, lymphatic permeation; HR, hazard ratio; PD-L1, programmed death ligand-1.

**Table 6:** Univariate and multivariate analysis of prognostic factors for overall survival.

Factor	Univariate analysis		Multivariate analysis	
	HR (95%CI)	*P* value	HR (95%CI)	*P* value
Age(>60 vs ≤60)	0.806(0.337-1.927)	0.618		
Gender(male vs female)	1.259(0.537-2.951)	0.587		
Differentiation(well vs moderate/poor)	0.553(0.201-0.519)	0.238		
Tumor size(>3cm vs ≤3cm)	2.650(0.945-7.437)	0.052		
Lymph node metastasis (yes vs no)	2.102(0.755-5.857)	0.138		
TNM stage (IV-III vs II-I)	0.505(0.201-1.274)	0.135		
Tumor location(rectum vs colon)	0.651(0.238-1.782)	0.390		
Ly(positive vs negative)	1.092(0.455-2.620)	0.841		
Vascular invasion(positive vs negative)	4.939(1.732-14.082)	0.001*	3.429(1.005-11.697)	0.049*
PD-L1 (positive vs negative)	5.142(1.673-15.805)	0.002*	4.267(1.144-15.917)	0.031*
SUVmax (high vs low)	2.385(0.968-5.876)	0.048*	1.229(0.393-3.847)	0.723
MTV3.0(high vs low)	3.935(1.504-10.298)	0.003*	1.456(0.142-14.881)	0.751
TLG3.0(high vs low)	3.757(1.448-9.743)	0.004*	1.578(0.140-17.758)	0.712

*P < 0.05 is considered statistically significant, calculated with continuous variable. CI, confidence interval; Ly, lymphatic permeation; HR, hazard ratio; PD-L1, programmed death ligand-1.
